# The Nashville Meeting

**Published:** 1897-10

**Authors:** 


### EDITORIAL.

### THE NASHVILLE MEETING.

THE last meeting of the United States Veterinary Medical Association was one of the largest and in all respects one of the best in the history of the organization. It was with some trepidation that many of the older members noted the decision to meet at the furthest point south reached by the Association, but nevertheless they turned out in force and did all they could for the meeting. It should not be thought that because the United States Veterinary Medical Association has never met so far south before that this vitiates in any degree its unchallenged claim to the title of a national organization. This, we believe, is the first instance in which it has been invited to a Southern city, and the members of its Executive Committee gladly accepted the invitation when it came. The Association has never met at points as far west as Denver or San Francisco, and this is largely because the veterinarians in these places and the veterinary organizations do not strive to obtain the meeting. Why should the veterinarians of any city or State or any local veterinary society endeavor to obtain a meeting of the United States Veterinary Medical Association in their respective localities? Ask Nashville, ask Tennessee. The South is the most rapidly growing portion of this country, and many portions of it have developed great industries and commercial enterprises that are adding rapidly to their wealth and general prosperity. The breeding of farm animals has not been neglected, and is gradually developing in harmony with the improved agricultural conditions. The people for some time have been awakening as to the necessity of a purer food-supply, and especially a sanitary supervision of meat and milk.

All of these influences have tended to create a demand for veterinary services and to open the way for veterinarians; but for some reason the recognition of the profession in the South has not been as rapid as in many other portions of the country. Perhaps this can be accounted for, in part, by the circumstances that the veterinary profession is so new in that region; that the advances that veterinary science has made during recent years have not received general recognition; that the value of veterinary science is not fully understood, and the fact that numerous colleges are turning out well-trained men, fully equipped to deal with the numerous intricate questions that come within the field of the veterinarian, has not been appreciated in that section. When the United States Veterinary Medical Association met in Nashville and drew to that city some of the most eminent pathologists and bacteriologists and men of science in the country—men of eminence, with national reputations; men of wide influence—the eyes of the people were opened. The newspaper press of the city reported the meeting fully and intelligently. A number of prominent sanitarians and breeders attended some of the sessions, and thus served still further to distribute the influence of the meeting.

It is safe to say that the Nashville meeting marks the beginning of a new era in veterinary progress in the South, provided the Southern veterinarians actively follow up and develop the impression that has been made.

The general character of the papers read and the discussion upon them was of a high order, and showed much careful research and diligent preparation.

The quality of veterinary work in this country is steadily improving under the leadership of the United States Veterinary Medical Association, and this is clearly indicated to the intelligent observer who studies the proceedings. These proceedings constitute a permanent record of the highest attainments of the general profession, hence it is important that they should be printed in a form that is alike creditable to the Association and the profession. It is pleasant to note that the Publication Committee, led by its active and energetic chairman, Dr. Williams, has promised the printed report within four weeks after the close of the session. The report is to be a full and accurate account of the meetings, to contain all of the papers and principal discussions, an official record of all the motions and resolutions, and is to be printed on good paper in a neat, workmanlike manner, fully equal to the present standard; and, crowning all, is to cost less than the amounts that have been expended for this purpose for a number of years. For the report of the minutiæ of the meeting we refer our readers to this publication, which will be in their hands by October 15th.

As a variation from the routine of scientific work, the election of officers occurred as a welcome diversion. The result of the election is most encouraging to all who are interested in the organization. As the meeting was held in the South, it was thought proper to elect a Southern man to the presidency. It was thought that this would be an appropriate act, and would tend to increase the influence of the organization in its new field. As a result of this sentiment, Dr. D. E. Salmon (whose legal residence is in Maryland) was elected to direct the Association during the coming year. Dr. Salmon has the true interests of the veterinary profession at heart, and, as chief of the Bureau of Animal Industry, is the leading veterinarian of the United States. Therefore, his selection by his Southern colleagues and other friends and admirers is a most happy one, and serves most agreeably and impressively to emphasize the real Southern character of the recent meeting. Another result of this election was to abolish the old-time precedent that an acceptable executive should be re-elected to a second term. It was felt that the present was an opportune time for this innovation, because Dr. Osgood's administration was placed beyond criticism by the energy and force which characterized his presidency; the progress of the organization under his direction has been so satisfactory, and the recommendations in his annual address so original, salient, and marked by universal approval.

The efficient Secretary, who has served the organization so faithfully for two years, was re-elected, and we are fortunate, indeed, in having Dr. Stewart again in charge of the vital matters under his care. Dr. Robertson, for the first time in years, failed to put in an appearance. It is needless to say that he was sadly missed, and the absence of his jovial smile and cheery words left a great vacancy. At his own request he was not re-elected to the post of Treasurer—one that he has filled so well for these many years—but was succeeded by that able and energetic young veterinarian, Dr. William Herbert Lowe, of Paterson, N. J.

As Vice-presidents, the Association has been successful in enlisting the services of Drs. T. B. Rayner, Rayen, and A. T. Peters.

The resolutions adopted by the Association, which will appear in another place in this JOURNAL, mark the trend of veterinary thought and effort at this time. Let all veterinarians read them carefully and use their utmost endeavors to further the ends indicated by them.

It is scarcely possible to express adequately the appreciation of the members for the entertainment provided by the local and the Southern veterinarians. The ladies, who were present in greater number than ever before, were taken care of by Mrs. Rayen and a corps of delightful assistants who entertained with true Southern hospitality.

As an echo of the recent meeting and a herald of the one that is to come, the question of the meeting-place for next year is prominent in the minds of all members of the organization. Boston, Washington, Omaha, and St. Louis are the candidates already in the field, and the claims of each are being advanced urgently. St. Louis and Omaha represent the middle West, Boston the East, and Washington the Southeast. As a reason for meeting in the West, it is claimed that the influence of the organization is needed there. As a reason for meeting in the East, it is held that as the meetings gyrated between Boston and New York for so many years, the veterinarians and local organizations of these places became accustomed to depend upon them for their inspiration and for the initiation of all new movements. Since the meetings have not been held in Boston for a number of years, a void has occurred, and the profession there is suffering in consequence. This question is one of the most weighty that will come before the new Executive Committee; but as our new President will undoubtedly appoint as members of this committee representatives from all of the important centres of veterinary work and life in the country, there can be no doubt that it will be settled in the way that will be best for the profession.

